# Re: Factors affecting recurrence and progression of high grade non invasive bladder cancer treated by intravesical BCG

**DOI:** 10.12669/pjms.306.6408

**Published:** 2014

**Authors:** Haichao Huang, Jie Jin, Xin Li

**Affiliations:** 1 Haichao Huang, Department of Urology,Peking University First Hospital and Institute of Urology, Peking University, National Urological Cancer Center, 8 Xishiku Street, Xicheng District, Beijing 100034, China.; 2 Jie Jin, Department of Urology,Peking University First Hospital and Institute of Urology, Peking University, National Urological Cancer Center, 8 Xishiku Street, Xicheng District, Beijing 100034, China.; 3 Xin Li, Department of Urology,Peking University First Hospital and Institute of Urology, Peking University, National Urological Cancer Center, 8 Xishiku Street, Xicheng District, Beijing 100034, China.

This retrospective study^1^ evaluated 64 patients treated with intravesical BCG for high grade NMIBC from Dec 2008 to July 2012. Variables such as smoking, tumor size, location and multiplicity were evaluated. The authors defined smokers with large and multiple high grade NMIBC as high risk group and early radical cystectomy (RC) was recommended. In this investigation, tumor location showed no significant association with the prognosis outcomes.

However, we conducted a long-term (median 131, 2-234 months) follow-up analysis including a total of 57 (19 female and 38 male, respectively) patients with T1G3 bladder cancer between 1994 to 2004. Variables such as age, number and size of tumors, tumor location, operation methods, recurrence, progression, tumor-specific survival (TSS) were evaluated. Tumor stage was assessed according to the Union for International Cancer Control (UICC) TNM classification of malignant tumors 2002. Tumor grade was assessed according to the WHO classification of 1973. All the patients had a transurethral bladder resection (TURB), among which 11 patients undertook RC within 3 months after TURB. All the TURB Specimens had a definite muscle layer. A total of 48 patients had at least 2 weeks of instillation chemotherapy, while only 4 patients undertook Bacillus Calmette Guerin. Multiple factors were simultaneously assessed using Cox proportional hazards analysis. P-values <0.05 were considered significant. Statistical analysis was completed using SPSS. We found that patients with tumor location in neck or trigone of the bladder had a worse progression and TSS rate. On both univariable and multivariable analysis showed statistical significance on progression (P= 0.003 and 0.0007) and TSS (P= 0.016 and 0.004, respectively).

In our series, progression was observed in 14 patient, among which 5(35.7%) patient were detected 5 years after the initial TURB. However, the mean follow up was 28.36 months in the investigation mentioned above. Therefore, we suppose the relatively short period of follow-up may reduce the predicting ability of tumor progression and TSS rate. Further researches with long term follow-up are needed. Finally, in our long-term analysis, tumors which were located in the bladder neck/ trigone showed worse progression and TSS rate.

## Note:

Reply to: Pak J Med Sci. 2014;30(2):326–330.

## References

[1] Khan MK, Ahmed I, Raza SJ (2014). Factors effecting recurrence and progression of high grade non invasive bladder cancer treated by intravesical BCG. Pak J Med Sci.

